# Prokaryotic communities of Indo-Pacific giant barrel sponges are more strongly influenced by geography than host phylogeny

**DOI:** 10.1093/femsec/fiy194

**Published:** 2018-10-04

**Authors:** T Swierts, D F R Cleary, N J de Voogd

**Affiliations:** 1 Marine Biodiversity, Naturalis Biodiversity Center, PO Box 9517, 2300 RA, Leiden, the Netherlands; 2 Institute of Environmental Sciences, Leiden University, PO Box 9518, 2300 RA, Leiden, the Netherlands; 3 Departamento de Biologia CESAM, Centro de Estudos do Ambiente e do Mar, Universidade de Aveiro, Aveiro, Portugal

**Keywords:** sponges, *Xestospongia*, microbiome, Indo-Pacific, coral reefs

## Abstract

Sponges harbor complex communities of microorganisms that carry out essential roles for the functioning and survival of their hosts. In some cases, genetically related sponges from different geographic regions share microbes, while in other cases microbial communities are more similar in unrelated sponges collected from the same location. To better understand how geography and host phylogeny cause variation in the prokaryotic community of sponges, we compared the prokaryotic community of 44 giant barrel sponges (*Xestospongia* spp.). These sponges belonged to six reproductively isolated genetic groups from eight areas throughout the Indo-Pacific region. Using Illumina sequencing, we obtained 440 000 sequences of the 16S rRNA gene V3V4 variable region that were assigned to 3795 operational taxonomic units (OTUs). The prokaryotic community of giant barrel sponges was characterized by 71 core OTUs (i.e. OTUs present in each specimen) that represented 57.5% of the total number of sequences. The relative abundance of these core OTUs varied significantly among samples, and this variation was predominantly related to the geographic origin of the sample. These results show that in giant barrel sponges, the variation in the prokaryotic community is primarily associated with geography as opposed to phylogenetic relatedness.

## Teaser

The prokaryotic communities of multiple giant barrel sponge species in the Indo-Pacific are more strongly influenced by geography than host phylogeny.

## INTRODUCTION

Sponges are among the oldest living multicellular animals and form symbiotic relationships with complex communities of microorganisms including archaea, bacteria and single-celled eukaryotes (Hentschel *et al*. 2012). These microbial symbionts are essential for the functioning and survival of marine sponges, and play key roles in processes such as CO_2_-fixation, nutrient cycling, secondary metabolite production and the conversion of dissolved organic matter into particulate organic matter (Schmidt *et al*. 2000; Fan *et al*. 2012; de Goeij *et al*. 2013, Zhang *et al*. 2015; Slaby *et al*. 2017). In high microbial abundance (HMA) sponges, microbes can make up 40% of the total weight (Friedrich *et al*. 2001). Cyanobacteria also provide more than half of the energy requirements of several sponge species by fixing carbon through photosynthesis (Wilkinson 1983). Due to this intricate relationship, sponges are often referred to as the 'sponge holobiont': the combination of the sponge host and all residing microorganisms (Webster and Thomas 2016; Pita *et al*. 2018).

Host species throughout the phylum Porifera often have characteristic microbial fingerprints (Thomas *et al*. 2016) and the differences among hosts can originate at an early reproductive phase (Schmitt *et al*. 2008). Certain microorganisms can be assimilated in gametes or other reproductive stages by the host sponge, and such vertical transmission ensures that essential bacteria, archaea and even yeasts are transmitted to their offspring (Ereskovsky, Gonobobleva and Vishnyakov 2005; Maldonado *et al*. 2005; Sharp *et al*. 2007; Funkhouser and Bordestein 2013). Another means of acquiring relevant microbes is through horizontal transmission, whereby microorganisms are recruited from the environment (Taylor *et al*. 2007; Sipkema *et al*. 2015). These recruits are often harvested from the rare biosphere and tend to be found at much greater densities within the sponge host (Lynch and Neufeld 2015). Recent studies have found that certain microbes deemed ‘sponge-specific’ may indeed be found in the surrounding seawater as well, albeit in very low abundances (Taylor *et al*. 2013). Hence, the seawater may act as a reservoir for these microbes, from which related sponges in distant geographic regions are populated through horizontal transmission (Moitinho-Silva *et al*. 2014).

Microbial host specificity and stability across time and space is potentially a derivative of co-speciation (Erwin *et al*. 2012; Hardoim *et al*. 2012; Webster *et al*. 2013; Pita *et al*. 2013; Cuvelier *et al*. 2014; Naim *et al*. 2014; Webster and Thomas 2016; Souza *et al*. 2017; Steinert *et al*. 2017). Related sponges from distant geographic regions can share microbial phylotypes that were not recorded in their respective non-sponge environments, suggesting that a common ancestor harbored these phylotypes and that they have been passed on by vertical transmission during speciation events into each lineage (Taylor *et al*. 2007; Lafi *et al*. 2009). Similar microbial fingerprints among more related host species does not, however, necessarily require coevolution (Moran and Sloan 2015). Certain substructures of the sponge host (such as pores, channels, choanocytes, etc.) could provide distinct microenvironments, which have allowed niche differentiation resulting in similar host species specificity patterns (Webster and Thomas 2016).

It is apparent that host identity shapes the microbial community of many sponges, and that in some cases geographic origin is also an important driver (Erwin *et al*. 2012; Schmitt *et al*. 2012; Pita, López-Legentil and Erwin 2013; Easson and Thacker 2014; Marino *et al*. 2017; Souza *et al*. 2017). However, it is hard to assess whether geography or phylogeny are equally important drivers, or that one of the two is more important. At present, there is a dearth of studies that incorporate both geography and phylogeny, especially at a large geographic scale and with large sample sizes. To pinpoint the relative importance of host identity and geography on the microbial community, research should be expanded to large sample sizes from closely related sponges with broad distributions and a similar bauplan. Such a study can also help to define the species-specific core microbiota. Generally, the core is defined as the operational taxonomic units (OTUs) present in most, or all, samples within a certain taxonomic level, and which exact definition is chosen usually does not alter the interpretation of the results (Turnbaugh *et al*. 2006; Huse *et al*. 2012; Otani *et al*. 2014; Walke *et al*. 2014; Astudillo-García *et al*. 2017). While the core microbiota of sponges as a whole has been elaborately discussed by Schmitt *et al*. (2012), the OTUs considered to be species-specific are based on one individual per species. Without replicates it is impossible to extrapolate which of the unique microbes occur in (almost) every specimen of that species, and are thus universal members of their microbiota.

Giant barrel sponges are a particularly suitable model for such research since they have a broad distribution on coral reefs around the globe and have an intricate phylogeny (Swierts *et al*. 2013, 2017). While three giant barrel sponge species have been described so far, namely*Xestospongia muta* from the Caribbean, *Xestospongia testudinaria* from the Indo-Pacific and *Xestospongia bergquistia* from the northeastern coast of Australia, molecular studies comparing these giant barrel sponge species were unable to find a separation that correlated with the species descriptions as they exist today (Setiawan *et al*. 2016a, Swierts *et al*. 2017). Recent studies have, furthermore, revealed that giant barrel sponges around the globe form a much broader species complex (Swierts *et al*. 2013, 2017; Bell *et al*. 2014; Setiawan *et al*. 2016b). Some of the species occur over large geographic areas, while others are confined to smaller water bodies, but a remarkable feature of this species complex is the lack of correlation between phylogenetic affinity and geography on global scales. While it is nearly impossible to distinguish among groups based on morphological characters, the sister group of each genetic group appears to occur in a different ocean. In other words, two visually similar individuals living one metre apart can be genetically more distinct from one another than from individuals living on a reef at the other side of the world (Swierts *et al*. 2017).

Previous studies on the giant barrel sponge microbiota found that they are dominated by *Chloroflexi*, Proteobacteria, Acidobacteria and Actinobacteria (Montalvo *et al*. 2005, 2014; Montalvo and Hill 2011; Polonia *et al*. 2014, 2017; Cleary *et al*. 2015; De Voogd *et al*. 2015). However, these studies included a small number of replicates and sites and used lower resolution sequencing methods. These restrictions hamper the ability to draw strong conclusions. Montalvo and Hill (2011) compared the microbiota of three *X. muta* specimens from a reef in Florida with three *X. testudinaria* specimens from a reef in Indonesia. They concluded that the bacterial communities associated with these sponges, although very similar, are highly specific to each of the species. However, since the sponges inhabit water bodies on opposite sides of the globe, it is hard to argue that the different microbial communities are a direct consequence of being two species, rather than being driven by their environments. On the other hand, Fiore, Jarett and Lesser (2013) found a significant effect of location on the symbiotic microbial communities in *X. muta*, but with the revelation of the existence of at least three giant barrel sponge species in the Caribbean, the differences linked to the environment could also be a consequence of sampling different cryptic species at different sites (Swierts *et al*. 2017). These examples illustrate the need to thoroughly examine how the microbial communities in giant barrel sponges vary with geography and phylogeny.

This is, to the best of our knowledge, the first study that includes intricate phylogenetic relationships within a single sponge genus at an ocean-wide scale in order to compare sponge microbiota. First, we characterize the core prokaryotic community within Indo-Pacific giant barrel sponges. Next, we test to what extent the variation in the prokaryotic community of giant barrel sponges can be explained by geography and host relatedness.

## METHODS

### Sample collection and study areas

**Figure 1.**
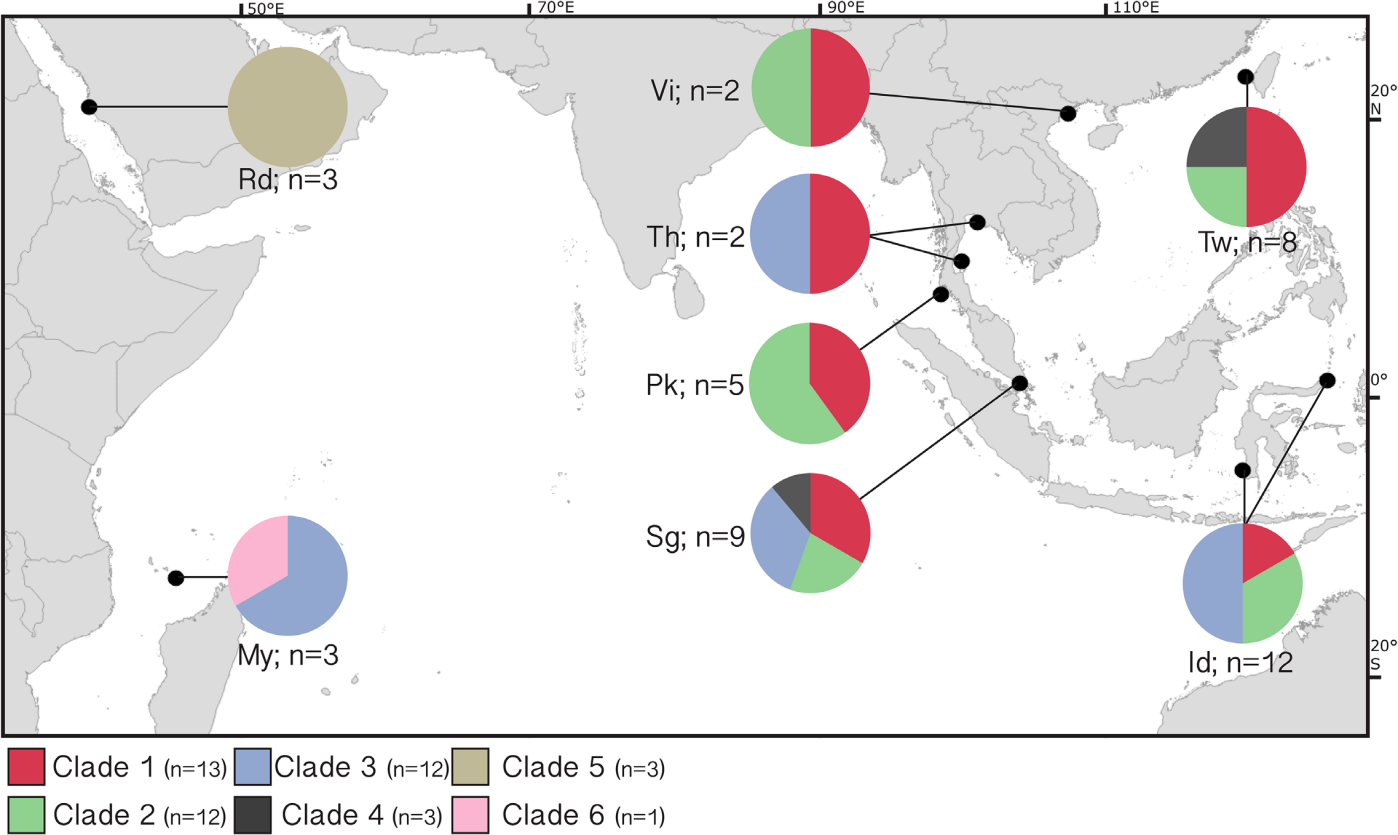
Map with the sampling sites per geographic region. Colors of the pie charts indicate the genetic clades of the sponge specimens. Abbreviations: Rd = Red Sea; My = Mayotte; Pk = Phuket, Thailand; Sg = Singapore; Th = Koh Tao and Pattaya, Gulf of Thailand; Vi = Vietnam; Id = Lembeh and Makassar, Indonesian seas; Tw = Taiwan.

Our dataset included 44 samples, unevenly collected by scuba diving from eight areas across the Indo-Pacific (Fig. 1). After collection, the material was immediately stored in absolute ethanol (98%) at -20°C. Sponge DNA extraction and the amplification of the mitochondrial genes CO1 and ATP6 were performed following the protocols described in Swierts *et al*. (2017).

For the 16S rRNA gene barcoded Illumina sequencing, we used the FastDNA SPIN Kit for Soil (MP Biochemicals) following the manufacturer's instructions. In brief, sponge samples were cut into small pieces containing both ectosome and choanosome, which were then added to a mixture of silica and ceramic particles in the manufacturer-provided Lysing Matrix E tubes. Cell lysis was performed in a Qiagen TissueLyser II during two sessions of 40 s at the maximum speed, with a 2-min interval between sessions to prevent the samples from overheating. Extracted DNA was eluted into DNase/Pyrogen-Free Water to a final volume of 40 μl and stored at −20°C until use.

### Clade delineation, distribution, codes and core

Recent studies have shown that what is currently considered *X. testudinaria* actually includes multiple reproductively isolated lineages (i.e. species) (Swierts *et al*. 2013; Bell *et al*. 2014; Swierts *et al*. 2017). In the absence of renewed species descriptions, we classified our samples into six clades, based on the CO1 and ATP6 mitochondrial genes, that correspond to the ‘groups’ or candidate species identified by Swierts *et al*. (2017). Some clades are found in different regions, with clade 3 being the most widespread with presence in the Indonesian Seas, Mozambique Channel, Gulf of Thailand and Singapore Strait (Fig. 1). Clades 5 and 6, on the other hand, are not widespread and are confined to the Red Sea and Mozambique Channel, respectively (Fig. 1).

Seven-symbol sample codes, as shown in certain figures and tables, contain the information of the location, clade and the sample number. The first two letters indicate the location (Pk = Phuket, Thailand; Rd = Red Sea; etc.), the next number indicates the genetic group (1 = clade 1; 2 = clade 2; etc.), and the following four symbols indicate the sample number (s001 = specimen 001; s004 = specimen 004; etc.). The location codes ‘Mk’ (Makassar) and ‘Lm’ (Lembeh) are both sublocations of ‘Id’ (Indonesian Seas).

While there is no consensus on which definition for the core microbiota should be used in sponges, limiting analyses to a core microbial community is a simple method to manage the complexity of the microbiota of marine sponges (Astudillo-García *et al*. 2017). In our analyses, we defined the core community as the sum of the OTUs present in every sponge specimen. This most stringent definition served as a good guideline, as our subject species are very closely related. However, changing the core definition of three species within the *Xestospongia* genus did not clearly influence the findings of beta-diversity (Astudillo-García *et al*. 2017).

### Sequence analyses

The 16S rRNA gene V3V4 variable region PCR primers 341F 5′-CCTACGGGNGGCWGCAG-3′ and 785R 3'-GACTACHVGGGTATCTAATCC-5' with barcode on the forward primer were used in a 28-cycle PCR assay (5-cycle used on PCR products) using the HotStarTaq Plus Master Mix Kit (Qiagen, USA) under the following conditions: 94°C for 3 min, followed by 28 cycles of 94°C for 30 s, 53°C for 40 s and 72°C for 1 min, after which a final elongation step at 72°C for 5 min was performed. After amplification, PCR products were checked in 2% agarose gel to determine the success of amplification and the relative intensity of bands. Multiple samples were pooled together in equal proportions based on their molecular weight and DNA concentrations. Pooled samples were purified using calibrated Ampure XP beads. Pooled and purified PCR product was used to prepare the DNA library following the Illumina TruSeq DNA library preparation protocol. Next generation, paired-end sequencing was performed at mrDNA Molecular Research LP (http://www.mrdnalab.com/; last checked 18 November 2016) on an Illumina MiSeq device (Illumina Inc., San Diego, CA, USA) following the manufacturer's guidelines. Sequences from each end were joined following Q25 quality trimming of the ends followed by reorienting any 3'-5' reads back into 5'-3', and removal of short reads (<150 bp). The resultant files were analyzed using the Quantitative Insights Into Microbial Ecology (QIIME) (Caporaso *et al*. 2010) software package (http://www.qiime.org/; last checked 20 January 2017).

In QIIME, fasta and qual files were used as input for the split_libraries.py script. Default arguments were used except for the minimum sequence length, which was set at 250 bps after removal of forward primers and barcodes. In addition to user-defined cut-offs, the split_libraries.py script performs several quality filtering steps (http://qiime.org/scripts/split_libraries.html). OTUs were selected using the UPARSE pipeline (https://www.drive5.com/usearch/manual7/uparse_pipeline.html; last checked 5 July 2018; Cleary *et al*. 2017; Cleary, Polónia and De Voogd 2018) with usearch10 (Edgar 2010). The UPARSE pipeline (Edgar 2013) includes clustering, chimera checking and quality filtering on de-multiplexed sequences. Chimera checking was performed using the UCHIME algorithm (Edgar *et al*. 2011). The quality filtering as implemented in usearch10 filters noisy reads and results suggest its output is comparable with other denoisers such as AmpliconNoise, but is much less computationally expensive (Edgar and Flyvbjerg 2015). First, reads were filtered with the -fastq_filter command and the following arguments: -fastq_trunclen 250, -fastq_maxee 0.5, -fastq_truncqual 15. Sequences were then dereplicated and sorted using the -derep_fulllength and -sortbysize commands. OTU clustering was performed using the -cluster_otus command followed by the -usearch_global command (using global alignment) with id set to 97% to map reads back to OTUs. AWK scripts were then used to convert the OTU files to QIIME format. In QIIME, representative sequences were selected using the pick_rep_set.py script in QIIME using the ‘most_abundant’ method. Taxonomy was assigned to reference sequences of OTUs using default arguments in the assign_taxonomy.py script in QIIME with the rdp method (Wang *et al*. 2007). In the assign_taxonomy.py function, we used a fasta file containing reference sequences from the SILVA 128 QIIME release and the uclust classifier method to map sequences to the assigned taxonomy. The make_otu_table.py script in QIIME was used to generate a square matrix of OTUs x SAMPLES followed by the single_rarefaction.py script to rarefy each sample to 10000 sequences. The rarefied table was used as input for further analyses using the R package (R Core Team 2013). We used the blastn command line tool in a Linux environment to query representative sequences of selected taxa including all of the most abundant (≥5000 sequences) OTUs against the online NCBI nucleotide database. Vectors were then generated containing sequence identifiers (GIs) of the 10 top hits of all representative sequences and the Entrez.efetch function in BioPython (Cock *et al*. 2009) was used with the retype argument set to ‘gb’ to download Genbank information of the aforementioned top hits including the isolation source of the organism and the host if relevant. The DNA sequences generated in this study can be downloaded from the NCBI SRA: SRP150943.

### Statistical analyses

A table containing the presence and abundance per sample of all OTUs was imported into R using the read.csv() function. Plant organelles, mitochondria, known contaminants (Salter *et al*. 2014) and sequences not assigned to a domain, phylum or class were removed prior to statistical analysis. Singletons were not removed in contrast to other studies, but the rigorous approach above and quality control steps during sequence analyses were taken to minimize the problem posed by sequencing errors in order to enable us to compare rare and abundant OTUs in our dataset. Pielou's J (H/log(S)) was calculated to estimate evenness using the diversity() function in the VEGAN package (Oksanen *et al*. 2016) in R. The OTU abundance matrix was log_e_ (*x* + 1) transformed (in order to normalize the distribution of the data) and distance matrices were constructed using the Bray-Curtis index with the vegdist() function in the VEGAN package. The Bray-Curtis index is one of the most frequently applied (dis)similarity indices used in ecology (Legendre and Gallagher 2001; Cleary 2003; Polónia *et al*. 2015, 2016). Variation in OTU composition was assessed with principal coordinates analysis (PCO) using the cmdscale() function in R with the Bray-Curtis distance matrix as input. We tested for significant variation among geography and phylogeny using an adonis() analysis. In the adonis analysis, the Bray-Curtis distance matrix of OTU composition was the response variable with geographical area and haplotype as independent variables. The number of permutations was set at 999; all other arguments used the default values set in the function. Weighted averages scores were computed for OTUs on the first two PCO axes using the wascores() function in the vegan package.

In order to test for phylogenetic differences between abundant and rare species we constructed two phylogenetic trees consisting of the two most abundant classes (*SAR202* and *Caldilineae*) of the *Chloroflexi*, which was the most abundant phylum in our study. For the purposes of this study, OTUs of the *Caldilineae* were considered abundant if they had >100 sequences in the total dataset. OTUs were considered rare if they had <5 sequences. For the *SAR202*, the numbers were >1000 sequences for abundant OTUs and <5 sequences for rare OTUs. With these cut-off values we obtained comparable amounts of ‘rare’ and ‘abundant’ OTUs per bacterial class. The ape (Paradis, Claude and Strimmer 2004), phangorn (Schliep 2011) and picante (Kembel *et al*. 2010) libraries were used during phylogenetic construction and analysis. First, fasta files containing representative sequences of abundant and rare OTUs were imported into R using the read.DNA() function. Sequences <350 bps were subsequently removed and the remaining sequences aligned using the muscle() function with arguments -gapopen -400.0, -gapextend -0.1, -seqtype dna and -cluster1 neighbor-joining. The resultant dataset was transformed using the as.DNAbin() function. The modelTest() function was used to compare different nucleotide or amino acid substitution models including tests for the Gamma model and invariant sites. The best model selection was based on Akaike information criterion (AIC) model selection (Akaike 1974). For all three classes the GTR + G + I model gave the best result. Neighbor-joining tree estimation (Saitou and Nei 1987) with the dist.hamming() function was achieved using the NJ() function with the ratio argument set to TRUE and the exclude set to pairwise. The resultant tree was analyzed using the pml() function, which computed the likelihood of the phylogenetic tree with the sequence alignment and GTR + G + I model. The number of intervals of the discrete gamma distribution was set to 4 and the proportion of invariable sites to 0.2. The optim.pml() function was subsequently used to optimize the different model parameters with the optNni, optGamma and optInv arguments all set to TRUE and the model argument set to GTR. Finally, the bootstrap.pml() function was used to perform bootstrap analysis on the resultant tree with the number of bootstraps set to 100 and other arguments following the optim.pml() function. All OTUs were assigned to either ‘abundant’ or ‘rare’ and the phylo.d() function in the package caper was used to calculate the D value, a measure of phylogenetic signal in binary traits, and to test for significant departure from random association. D values of 1 indicate random association while D values <1 indicate clumping and values >1 indicate overdispersion. Detailed descriptions of the functions used here can be found in R (e.g. ?cmdscale) and online in reference manuals (http://cran.r-project.org/web/packages/vegan/index.html).

## RESULTS

### Core microbiota

Illumina sequencing of the 16S rRNA gene V3V4 variable region from 44 giant barrel sponges throughout the Indo-Pacific yielded 440 000 sequences. These sequences were assigned to 3795 OTUs after quality control. The OTUs were assigned to 48 phyla, 106 classes and 145 orders. *Proteobacteria* was the most diverse and abundant phylum with 134 057 sequences from 1541 OTUs. *Chloroflexi* were almost equally abundant with 126 358 sequences, but with 448 OTUs they were less diverse than *Proteobacteria*. Other diverse phyla included *Bacteroidetes* (239 OTUs), *Acidobacteria* (178), *Actinobacteria* (171), *Gemmatimonadetes* (163), *Planctomycetes* (134), *Cyanobacteria* (111) and *Poribacteria* (62).

According to our definition, the core consisted of 71 OTUs (1.9% of all OTUs) which together yielded 252 988 sequence reads (57.5% of the total number of sequences) (Table S1; see the supplementary data). Hence, a small number of OTUs make up the majority of the giant barrel sponge microbiota, illustrating the core's importance. In our dataset of healthy Indo-Pacific giant barrel sponges, 38–69% of the sponge microbiota consisted of OTUs present in all giant barrel sponges. The sample with the lowest relative contribution of its core community (38.8%) was a sponge from Taiwan (Tw4s476) and the sample with the highest relative contribution of its core community (68.6%) was a sponge from Lembeh, Indonesia (Lm3s005).

The most diverse phylum in the core community was *Chloroflexi* (25 OTUs), which included two members of the class *Caldilineae* and 18 members of the class *SAR202*. Whereas the most abundant core OTU was a member of the *Caldilineae* (OTU 1; 17 592 sequences; 7% of the total amount of core sequences), the *SAR202* members combined added up to 23.2% of the total core sequences and were the most abundant bacterial class in the giant barrel sponge core. Other phyla in the core were *Proteobacteria* (19 OTUs), *Actinobacteria* (7), *Gemmatimonadetes* (5), *Acidobacteria* (4), *Nitrospirae* (2) and *Poribacteria*(1). No archaeon was part of the core prokaryotic community; however, each giant barrel sponge harbored at least one OTU from the archaeal genus *Candidatus Nitrosopumilus*.

Nearly half of the OTUs (49.9%) occurred in only one sponge individual, and many of these OTUs returned only one sequence read. The OTUs occurring in one specimen encompassed only a small proportion of the total amount of sequence reads (0.48%).

### Host specificity compared to geography and host phylogeny

**Figure 2.**
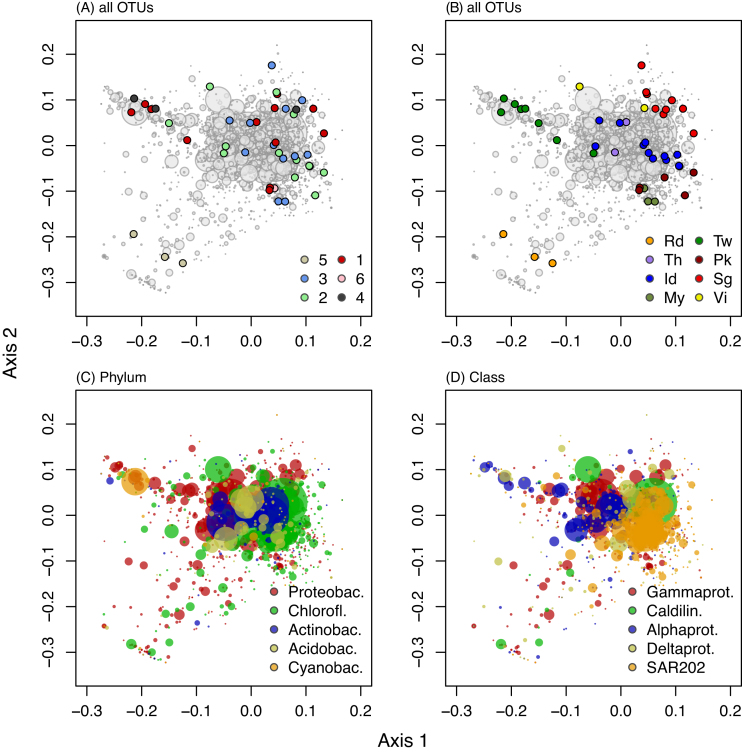
First and second axes of the Principle Coordinate Ordination based on our full dataset. Each dot in the (**A**) and (**B**) graphs represents one sponge individual, and their positioning in the ordination is identical for both (A) and (B), the only difference being the color scheme. Colors in (A) indicate clades and in (B) geographic origin. Abbreviations of geographic locations are: Rd = Red Sea; My = Mayotte; Pk = Phuket, Thailand; Sg = Singapore; Th = Koh Tao and Pattaya, Gulf of Thailand; Vi = Vietnam; Id = Lembeh and Makassar, Indonesian seas; Tw = Taiwan. The OTUs are color-coded for phylum in (**C**) and bacterial class in (**D**).

The results of our PCO analysis, based on all 3795 OTUs, are shown in Fig. 2. The samples visually cluster together based on geography. Samples from the Gulf of Thailand, Indonesia, Mayotte, Phuket and Singapore are separated along the first PCO axis from samples from the Red Sea and Taiwan. This axis explained 19.7% of the variation in our PCO analysis. The second axis, which explained 13.3% of the variation, separated the sponges of clade 5, which were all collected in the Red Sea, from the other clades and locations. The third and fourth axes, which explained 8.0% and 6.2% of the variation, respectively, followed the same pattern, with samples clustering based on geography rather than phylogeny (Figure S2; supplementary data). Both geography (adonis: F_5,41 _= 3.00, *P* < 0.001, R^2^ = 0.368) and phylogeny (adonis: F_5,41 _= 1.86, *P* < 0.001, R^2^ = 0.197) were significant predictors of variation in the composition of the prokaryotic community. Due to the larger influence of geography, and the lack of obvious clustering in our PCO analysis based on phylogeny, we focused on the variation in prokaryotic communities of giant barrel sponges with regard to geography in subsequent analyses.

**Figure 3.**
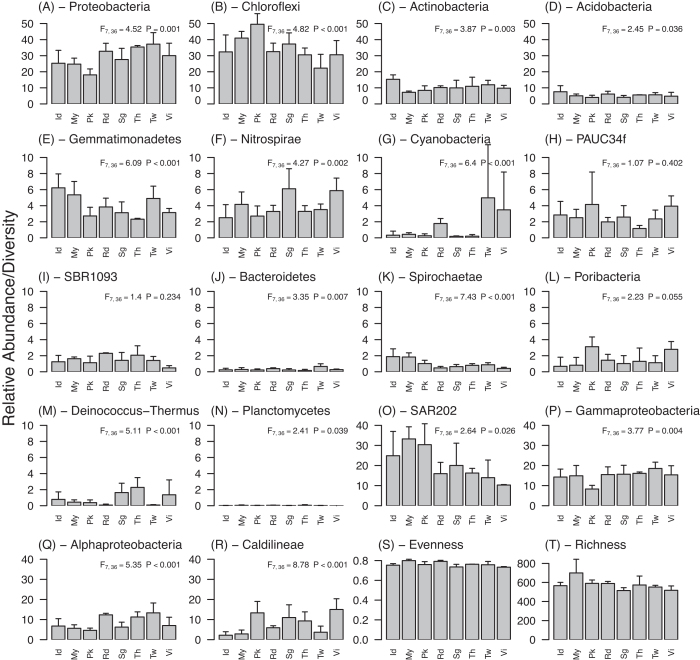
Mean relative abundance of all OTUs within the most abundant bacterial phyla (**A**-**N**) and classes (**O**-**R**) and the evenness (**S**) and richness (**T**) for giant barrel sponges from eight locations around the globe (Rd = Red Sea; My = Mayotte; Pk = Phuket, Thailand; Sg = Singapore; Th = Koh Tao and Pattaya, Gulf of Thailand; Vi = Vietnam; Id = Lembeh and Makassar, Indonesian seas; Tw = Taiwan). Error bars indicate the standard deviation. Results of GLM (General Linear Model) are shown in the top right corner of each graph.

The abundance of some higher bacterial taxa among geographic locations varied significantly (Fig. 3). The Red Sea, Gulf of Thailand, Taiwan and Vietnam were characterized by relatively high numbers of *Proteobacteria*and low numbers of *Chloroflexi*, while the opposite was true for sponges from the Indonesian Seas, Mayotte, Phuket and Singapore (Fig. 3a,b). The abundance of the phyla *Actinobacteria*, *Acidobacteria*, *Gemmatimonadetes, Nitrospirae, Cyanobacteria, Bacteroidetes, Spirochaetae, Deinococcus − Thermus* and *Planctomycetes* differed significantly among groups from different geographic regions (Fig. 3c-g,j,k,m,n). In contrast, *PAUC34f, SBR1093* and *Poribacteria* did not show a similar effect (Fig. 3h,i,l). In addition to phyla, certain bacterial classes also differed significantly among locations (Fig. 3o-r). For example, the bacterial classe*s SAR202*and*Caldilineae*showed a large variation in relative abundance, varying from 10.3 (± 3.6)% in Vietnam to 30.1 (± 5.0)% in Mayotte for *SAR202*, and from 1.9 (± 1.6)% in the Indonesian Seas to 12.7 (± 5.9)% in Phuket for *Calidilineae* (Fig. 3o,r). For these two bacterial classes, we tested whether abundant OTUs were phylogenetically related to one another. We found a significant phylogenetic clumping of abundant OTUs within the *Caldilineae* (estimated D: 0.365; *P* < 0.001), whereas this was not observed for *SAR202* (estimated D: 1.583; *P* = 1.000), where abundant OTUs did not cluster together in the phylogenetic tree (Figure S3; supplementary data). The evenness and rarefied richness per geographical location are shown in Fig. 3s,t.

The abundance of certain individual OTUs was also related to geography. The most abundant OTU (OTU 1; 15 592 sequences) in our dataset was assigned to the family *Caldineaceae* within the *Caldilineae*, and was similar to an organism previously found in giant barrel sponges from Indonesia (sequence similarity = 100%; Table S4; supplementary data). Although this was the most abundant OTU in our total dataset, there was pronounced variation in its relative abundance among geographic locations, varying from an average abundance of <1% in Taiwan (0.72 ± 0.69%) to 12% in Phuket (11.86 ± 5.03%).

The second most abundant OTU in our dataset (OTU 2; 11 491 sequences) was assigned to the class *Nitrospira* and was closely related to an organism found in the coral *Porites lutea* (sequence similarity = 100%; Table S4; supplementary data). This OTU was most abundant in sponges from Singapore (4.0 ± 3.1%) and Vietnam (5.4 ± 1.35%), and it was often the dominant *Nitrospira* member in the giant barrel sponge microbiota with very low numbers of other OTUs assigned to the *Nitrospira* (Fig. 3).

The third most abundant OTU in our dataset (OTU 3; 18 996 sequences) was assigned to the class *SAR202*, within the *Chloroflexi*, and was closely related to an organism previously found in the sponge *Astrosclera willeyana* (Table S4; supplementary data). Each giant barrel sponge sample hosted a fair number of sequences of OTU 3 (47–598 reads), but simultaneously also harbored a rich variety of 15 to 58 OTUs of other moderately abundant *SAR202* members (>0.1%). One sponge from Phuket, Thailand (Pk2s085) even harbored 16 OTUs of *SAR202* which each comprised at least 1% of its total community. This is different to the previously mentioned classes, *Caldilineae* and *Nitrospira*, in which one specific OTU of each of the respective bacterial classes was often abundant.

**Figure 4.**
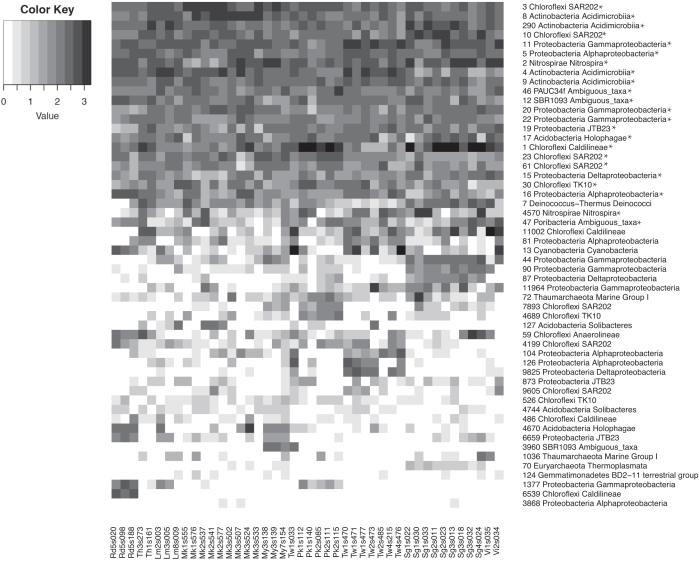
Heat map indicating the abundance of the 19 most abundant OTUs in our dataset and 35 handpicked OTUs in each giant barrel sponge sample. The handpicked OTUs are specified in Table S4. The sponges are ordered based on geography (Rd = Red Sea; My = Mayotte; Pk = Phuket, Thailand; Sg = Singapore; Th = Koh Tao and Pattaya, Gulf of Thailand; Vi = Vietnam; Lm and Mk = Lembeh and Makassar, Indonesian seas; Tw = Taiwan) and clade (numbers 1–6 after geography code). Scale is logarithmic. Asterisks indicate OTUs that are part of the core (i.e. OTUs present in each sample in our dataset).

Fig. 4 illustrates that some OTUs were strongly restricted to specific locations. The OTUs included in this graph were selected because their presence varied with location. For example, OTU 3960 was predominantly found in samples from Mayotte. This OTU was assigned to the bacterial class *EC214*, and is related to a bacterium previously found in a sponge from the Red Sea (sequence similarity = 99.56%; Table S4; supplementary data), but remarkably enough this OTU is completely absent in our Red Sea samples. In Mayotte, the relative abundance of this OTU is 0.96 ± 0.26%, and besides being present in one Taiwanese specimen, it was virtually absent in all other sponges.

The Red Sea also had a distinct prokaryotic community. OTU 6539 made up 1.0–3.0% of the bacterial community of these specimens, but was nearly absent in all other samples (Fig. 4). It was related to an organism obtained from *Ircinia strobilina* in Bahamian mangroves (sequence similarity = 99.53%; Table S4; supplementary data). Other characteristic OTUs for the Red Sea are the OTUs 1377, 4670 and 6659 (Fig. 4; Table S4; supplementary data). These specific OTUs, together with the high relative abundances of *Alphaproteobacteria* and *Cyanobacteria* (Fig. 3), give the Red Sea a distinct prokaryotic community as evidenced by the distinct cluster it forms in the PCO analysis (Fig. 2). Since all Red Sea samples belonged to clade 5, a clade that was not found in other locations, this distinct Red Sea prokaryotic community is likewise characteristic for clade 5.

## DISCUSSION

### Core microbiota

Focusing on a core microbiota is a straightforward approach to manage the complexity of the microbiota of marine sponges (Astudillo-García *et al*. 2017). The prokaryotic community of giant barrel sponges in the Indo-Pacific is characterized by a relatively high number of core OTUs (i.e. OTUs present in each specimen) that represent the majority of the total number of sequences. In five other sponge species, both LMA (Low Microbial Abundance) and HMA (High Microbial Abundance), the core microbiota varied between seven and 20 OTUs, with each of those OTUs present in at least 85% of the samples (Thomas *et al*. 2016). With our more stringent definition of a core OTU, we found that Indo-Pacific giant barrel sponges have a diverse core, with 71 OTUs occurring in each specimen. The main bacterial phyla in the core prokaryotic community were *Proteobacteria, Chloroflexi, Actinobacteria, Gemmatimonadetes, Nitrospirae, Acidobacteria, PAUC34f* and *Poribacteria*. Members of *Chloroflexi* have been shown to be capable of harvesting energy from sunlight (Bryant and Frigaard 2006). The fact that 31 OTUs assigned to the *Chloroflexi* coexist in each giant barrel sponge in our Indo-Pacific dataset suggests that the giant barrel sponge holobiont is mixotrophic, and that photosynthesis may be an important pathway in its physiology. The same bacterial phyla were also among the main groups found in previous studies of the microbiota of giant barrel sponges (Montalvo and Hill 2011; Fiore, Jarett and Lesser 2013; Morrow *et al*. 2016; Cleary *et al*. 2015; De Voogd *et al*. 2015; Astudillo-García *et al*. 2017). Previously, members of the *Actinobacteria* were suggested to dominate the microbiota of *X. muta*, making up 12% of the community based on clone libraries (Montalvo *et al*. 2005). In line with Olson and Gao (2013), and Morrow *et al*. (2016), our data indicates that they are not the largest group in the microbiota; however, they are still an important contributor to the prokaryotic community, particularly in absolute numbers of sequences.

Core OTUs may possess traits that are beneficial for the host's survival in the Indo-Pacific since they occur in all sampled giant barrel sponges irrespective of their geographical origin or phylogenetic position. To determine which of these OTUs are fundamental for the giant barrel sponge species complex as a whole, these core OTUs should be compared with those of giant barrel sponges from other locations not included in this study, particularly the Caribbean and Australia. For example, a BLAST search of one OTU returned an identical sequence from a Caribbean giant barrel sponge (Montalvo and Hill 2011). The associations with OTUs that are specific to giant barrel sponges, and that occur in each specimen around the globe, may have originated in a common sponge ancestor prior to the first speciation event, whereas the associations with OTUs that are only found in all Indo-Pacific specimens but not necessarily in specimens from the other locations may have co-diversified locally with the giant barrel sponge species complex after the first speciation events.

In contrast to the core OTUs, a large number of OTUs only occurred in a single individual sponge. Almost half of the OTUs were such singularly occurring OTUs and should therefore not be considered specific to giant barrel sponges in general. Host species specificity implies that the OTU is characteristic for sponges of a certain species, but this is not the case for these singularly occurring OTUs. They are potentially misleading in the interpretation of interspecies comparisons as they might be mistaken for host-specific OTUs, particularly when the comparisons are based on just one sample or only a few samples per host species. It is likely that the number of 70% of host-species specific OTUs that was identified by Schmitt *et al*. (2012) is an overestimation, since this number probably contains such OTUs that were only found in one individual.

### Host specificity compared to geography and host phylogeny

Previously, it was found that prokaryotic communities of sponges are generally stable across sampling events, seasonal shifts in temperature and irradiance, and across large spatial scales (Erwin *et al*. 2012; Björk *et al*. 2013; Reveillaud *et al*. 2014; Steinert *et al*. 2016; Thomas *et al*. 2016). This was also true for giant barrel sponges (Olson and Gao 2013; Morrow *et al*.^2016^), but our results have led us to a different interpretation. The relative abundance of core OTUs and non-core OTUs varied considerably, and this variation was mostly related to the geographic origin of the sample, and to a lesser extent to the phylogeny. Samples from the same location had very similar prokaryotic communities, irrespective of the present genetic clades. In more isolated regions, such as the Red Sea and Mayotte, the sponges harbored specific OTUs that were orders of magnitude more abundant compared with sponges from other locations. In contrast to the Red Sea, multiple clades occur in Mayotte, and therefore the specificity of certain OTUs to several locations seems to be related to geography rather than phylogeny. In addition to giant barrel sponge-specific OTUs, one could argue that geography-specific OTUs within giant barrel sponges also exist.

The giant barrel sponge microbiota is believed to play key roles in nutrient cycling, and these communities may adapt to local light conditions and nutrient availability (Webster and Taylor 2012; Morrow *et al*.^2016^). Not all bacterial phyla and classes varied in a similar fashion or magnitude across the sampled locations. The groups that varied stronger, for example *Chloroflexi*, *Cyanobacteria* and *Nitrospirae*, might be more sensitive to local or regional environmental factors than other microbial groups with a more uniform distribution across the various areas. Many members of the class *SAR202* within the *Chloroflexi*, for example, are associated with sulphite oxidation in aphotic conditions, and this could be an important function in certain populations of giant barrel sponges depending on the local conditions (Mehrshad *et al*. 2018). Other studies have also found that the abundances in the sponge microbiota of several bacterial groups may correlate with environmental factors such as depth, turbidity, available food sources, pH and temperature (Olson, Thacker and Gochfeld 2014; Luter *et al*. 2015; Morrow *et al*. 2015; Lesser, Fiore and Slattery 2016). The geographical variation in the giant barrel sponge microbiota is not a direct derivative of the local microbiota from the abiotic environment, since it has been shown that both the bacterial and archaeal communities of both sediment and seawater are highly dissimilar to the prokaryotic community of giant barrel sponges (Polónia *et al*. 2014; Cleary *et al*. 2015; De Voogd *et al*. 2015).

While giant barrel sponges from the same location harbored more similar prokaryotic communities compared with giant barrel sponges from locations further away, phylogenetic relationships were also, albeit to a lesser extent, a predictor of prokaryotic community composition. However, these results were not visually detectable in the PCO analysis. This could simply be overshadowing of the phylogenetic signals by the stronger geographic signals in the analysis. However, this could also be the result of the genetic groups not being equally distributed over the geographic locations. For instance, all samples from the Red Sea belonged to one clade that was unique for that location (Swierts *et al*. 2017). The significant phylogenetic signal in our statistical test could, therefore, be a type I error as a result. This makes it difficult to confirm or reject hypotheses regarding the influence of phylogeny on the giant barrel sponge prokaryotic community.

Our results contradict the conclusions of a previous study comparing the microbiota of *X. muta* from Florida with *X. testudinaria* from Indonesia (Montalvo and Hill 2011). In this study, the authors concluded that the differences between the two species suggested vertical transmission and bacterial speciation within sponge hosts. However, after the recently exposed intricate and intertwined phylogenies of Caribbean and Indo-Pacific giant barrel sponges, it has become clear that the *X. testudinaria* samples used in their study were actually two different species (clade 1 and clade 3; Setiawan *et al*. 2016a; Setiawan *et al*. 2016b; Swierts *et al*. 2017). Therefore, it is more likely that the differences in the microbial communities reflect the geographic locations they were sampled in. Some of the lineages within the giant barrel sponge species complex are suggested to have been diverging since a time before the closing of the Tethys Seaway, approximately 50 million years ago (Swierts *et al*. 2017). Nevertheless, while these clades have genetically grown apart for millions of years, the sponges have retained nearly identical body plans. This taxonomical similarity may have allowed prokaryotic lineages to move from one giant barrel sponge clade to another by horizontal transmission, limiting or preventing co-diversification between prokaryotes and individual giant barrel sponge species (Moran and Sloan 2015).

Whether the giant barrel sponge prokaryotic community composition adapts to local conditions, or that available OTUs in the surrounding seawater are driving the variation, remains unknown. This study, however, shows that the environment can be a more important driver of the prokaryotic community than is generally considered. Furthermore, this study underlines the importance of incorporating geographic variation in comparisons among the prokaryotic communities of multiple sponge species or taxa.

## Supplementary Material

Supplementary Data
