# Supplementary material for: Prokaryotic communities of Indo-Pacific giant barrel sponges are more strongly influenced by geography than host phylogeny
Source: FEMS Microbiol Ecol. 2018 Oct 4;94(12):fiy194. doi: 10.1093/femsec/fiy194 (PMC6196991; doi:10.1093/femsec/fiy194)
Supplement: Supplementary Data [file fiy194_supplemental_files.zip › Supp 3.docx]

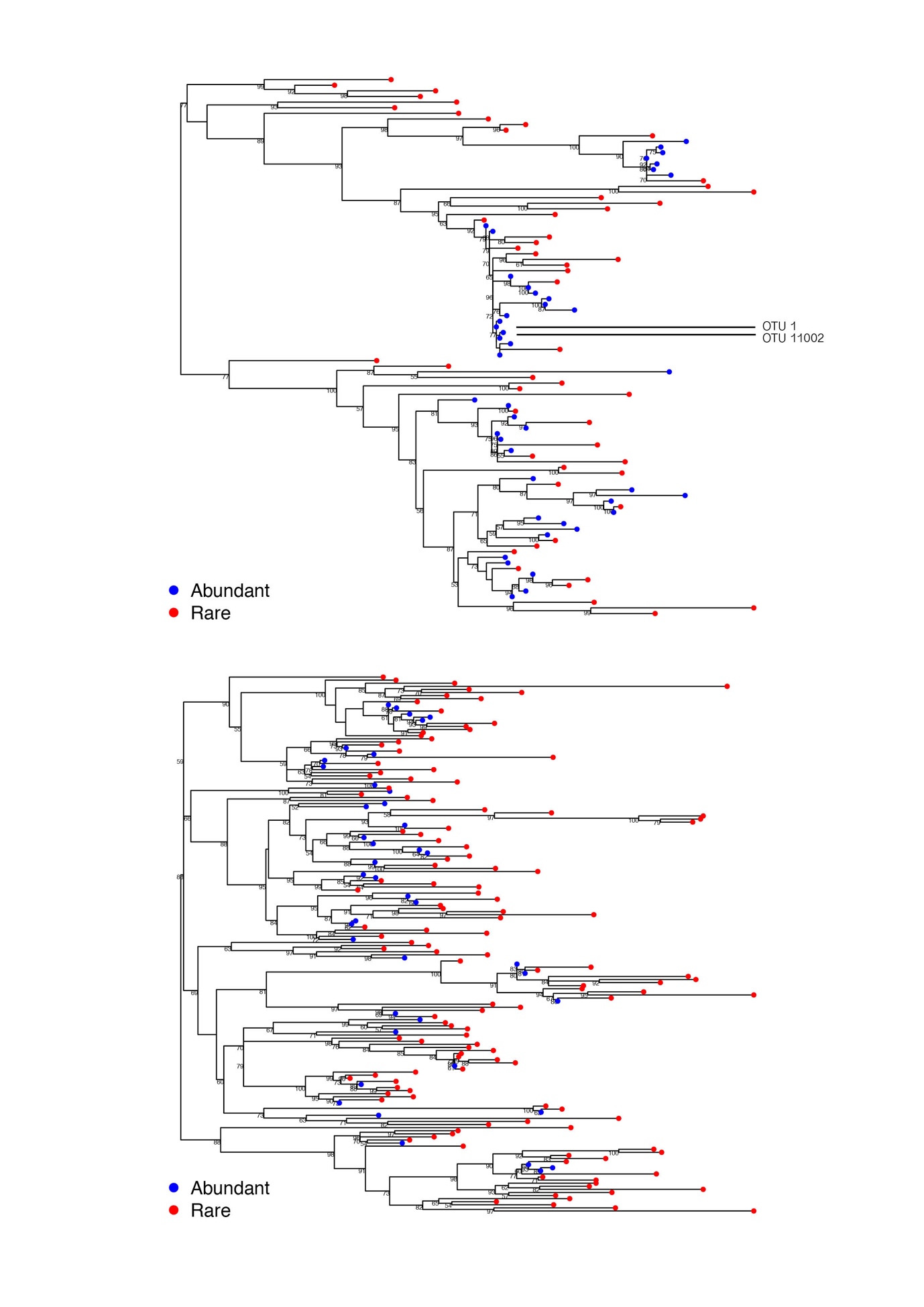


**Supporting Information 3.** Phylogenetic trees of the OTUs from bacterial classes Caldilineae (top) and SAR202 (bottom). Colours at the end of the branch indicates the abundance of the OTU: Red = Rare; Blue = Abundant. OTUs of the Caldilineae were considered abundant if they had > 100 sequences in the total dataset. OTUs were considered rare if they had < 5 sequences. For SAR202, OTUs were considered abundant if they had > 1000 sequences and rare if they had < 5 sequences. With these cut-off values we obtained similar amounts of 'rare' and 'abundant' OTUs per bacterial class.
