# Supplementary figures and images for: Prokaryotic communities of Indo-Pacific giant barrel sponges are more strongly influenced by geography than host phylogeny
Source: FEMS Microbiol Ecol. 2018 Oct 4;94(12):fiy194. doi: 10.1093/femsec/fiy194 (PMC6196991; doi:10.1093/femsec/fiy194)

a. all OTUs

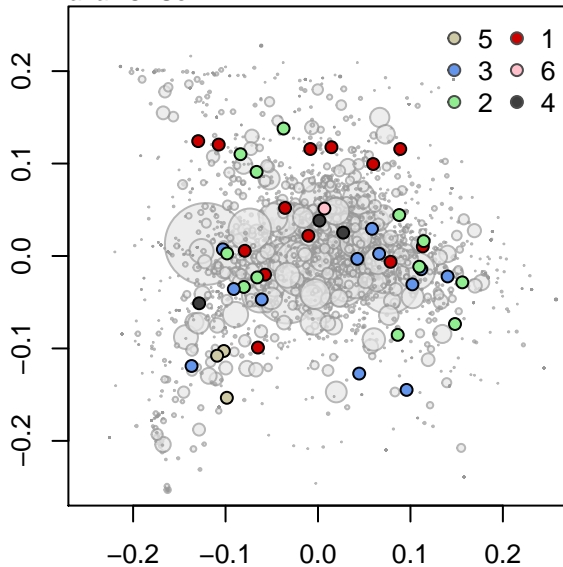

b. all OTUs

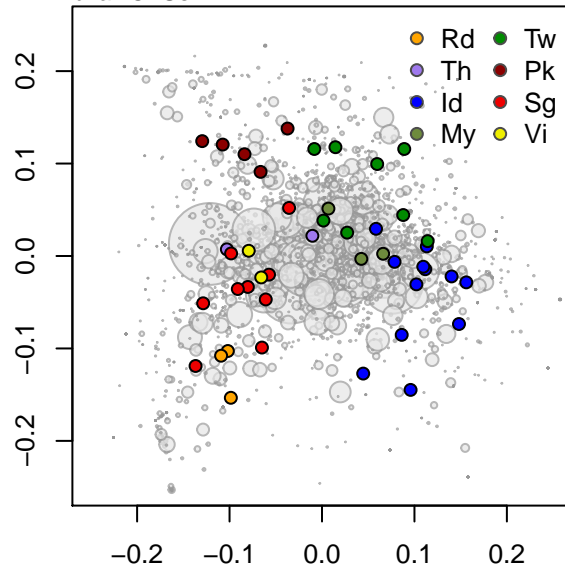

c. Phylum

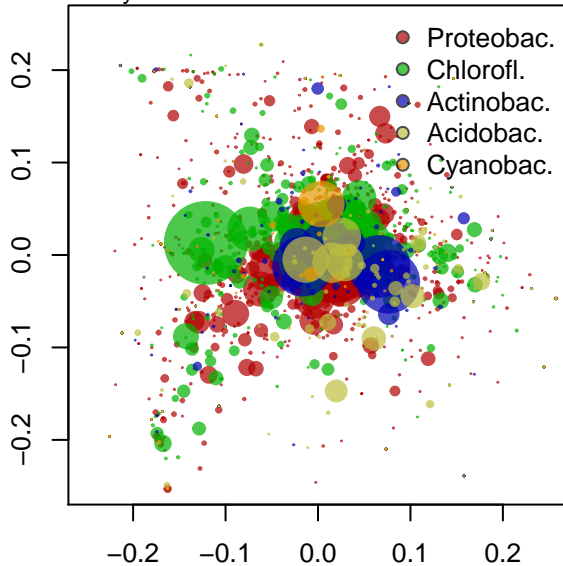

d. Class

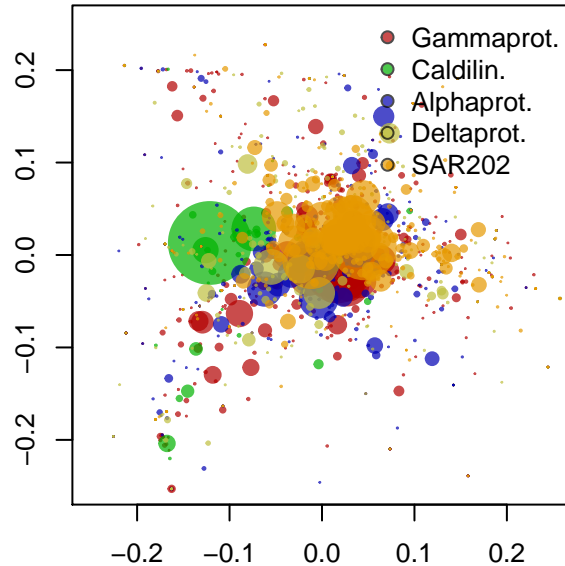

Axis 4

Axis 3

Supplement: Supplementary Data [file fiy194_supplemental_files.zip › Supp 2.pdf]
